# Overexpression of BMPER in Ovarian Cancer and the Mechanism by which It Promotes Malignant Biological Behavior in Tumor Cells

**DOI:** 10.1155/2020/3607436

**Published:** 2020-03-24

**Authors:** Yong Xi, Xin Nie, Jing Wang, Lingling Gao, Bei Lin

**Affiliations:** ^1^Department of Obstetrics and Gynecology, Shengjing Hospital Affiliated to China Medical University, Liaoning, China; ^2^Key Laboratory of Maternal-Fetal Medicine of Liaoning Province, Key Laboratory of Obstetrics and Gynecology of Higher Education of Liaoning Province, Liaoning, China; ^3^Dalian Municipal Maternal and Child Health Care Hospital, Liaoning, China

## Abstract

**Background:**

BMPER has been reported to be associated with the biological behavior of a few malignant tumors, but the mechanism is still unclear. We aimed to detect BMPER expression in ovarian epithelial tumor tissues and its effects on their biological behaviors, as well as to elucidate the possible mechanism.

**Methods:**

BMPER expression in ovarian epithelial tumor tissues was detected by immunohistochemistry. BMPER expression in ovarian cancer cell lines was inhibited via RNA interference. Changes in the malignant behaviors of ovarian cancer cells were detected by MTT, wound healing, Transwell, and flow cytometry assays. Changes in proteins in the MAPK and autophagy-related signaling pathways were detected by Western blot analysis.

**Results:**

The expression of BMPER was significantly upregulated in ovarian epithelial malignant tumors and was related to increased lymph node metastasis and lower survival rate. High BMPER expression is an independent risk factor for poor prognosis in patients. Inhibition of BMPER inhibited the proliferation, invasion, and migration of ovarian cancer cells and promoted apoptosis. In addition, BMPER downregulation decreased the expression of PCNA, Bcl-2, MMP2, and MMP9 and increased the expression of Bax. Moreover, the levels of p-ERK, p-MEK, and the autophagy-related protein p-mTOR were decreased, and Beclin 1 levels and the LC3II/I ratio were increased.

**Conclusions:**

Our findings indicated that BMPER is closely related to poor prognosis in ovarian cancer. BMPER plays a role in promoting the malignant biological behavior of tumor cells through the MAPK and autophagy-related signaling pathways.

## 1. Introduction

Epithelial ovarian cancers are the most common type of ovarian malignancy in female reproductive organs. Their mortality rate is high due to the absence of obvious symptoms during the early stages and a lack of reliable screening methods and specific biomarkers [1]. Although the five-year survival rate of patients with stage I ovarian cancer has been reported to be greater than 90%, patients with advanced stage disease have a five-year survival rate of only 29% [2]. Therefore, it is necessary to explore new valuable biomolecular markers to improve the diagnostic accuracy of early ovarian cancer and to reflect disease progression before and after treatment.

Bone morphogenetic protein (BMP) endothelial cell precursor-derived regulator (BMPER) was originally found in screens for differentially expressed proteins in embryonic endothelial precursor cells [3]. BMPER is a secretory glycoprotein expressed by endothelial precursor cells and is highly expressed in the lung, brain, prostate, appendix, and liver of adults. It plays an important role in normal embryonic growth and development and participates in regulating the development and physiological functions of various organs in the body. Moreover, it is associated with the occurrence or progression of various diseases, such as diaphonospondylodysostosis (DSD), pneumonia, pulmonary fibrosis, and tumors [4–6]. Currently, BMPER has been proven to promote the biological behaviors of some malignant tumor cells, such as lung cancer and colon cancer cells, but the specific mechanism underlying its role in tumors is not completely clear [7]. Currently, no reports have indicated the role of BMPER in ovarian cancer.

The mitogen-activated protein kinase (MAPK) pathway is a ubiquitous signal transduction pathway in eukaryotes and is involved in regulating various cellular physiological and pathological processes such as proliferation, apoptosis, invasion, migration, cell cycle progression, and differentiation. The MAPK signaling pathway nodal protein p-ERK1/2 is expressed in both ovarian cancer tissues and cells. Downregulation of p-ERK expression in ovarian cancer cells can inhibit their proliferation and induce apoptosis and G0/G1 phase arrest, suggesting that the MAPK pathway may play a key role in the occurrence and progression of ovarian cancer [8]. The results of our previous research showed that the MAPK signaling pathway was associated with malignant biological behaviors of epithelial ovarian cancer cells and that MAPK pathway inhibitors can inhibit the proliferation, invasion, and migration of ovarian cancer cells [9].

Autophagy has a dual role in malignancies, including ovarian cancer, which is related to the tumor type, microenvironment, treatment methods, and genetic factors [10]. Autophagy-related signaling pathways in ovarian cancer are usually downregulated, which exerts self-protective effects in ovarian cancer tissues. In this case, promoting autophagy can inhibit the progression of ovarian cancer. Autophagy induction is closely related to the promotion of apoptosis and inhibition of proliferation in ovarian cancer [11]. Autophagy inhibition promotes the invasion and migration of ovarian cancer cells A2780 and SKOV3 [12].

The aim of our work is to elucidate the role and mechanism of BMPER in the occurrence and progression of ovarian cancer and to explore whether BMPER affects the malignant biological behavior of ovarian cancer cells through the MAPK and autophagy-related signaling pathways in order to provide a valuable theoretical basis for screening and monitoring as well as predicting the prognosis of early ovarian cancer.

## 2. Materials and Methods

### 2.1. Sample Source and Clinical Data

A total of 171 paraffin-embedded ovarian tissue samples were obtained from surgical tissue from 2008 to 2014 in the Department of Gynecology of Shengjing Hospital of China Medical University. All histological sections were reviewed by pathologists in our hospital to confirm the diagnosis. The specimens were divided into four groups based on the tissue type: 94 ovarian epithelial malignant tumors (malignant group), 24 ovarian epithelial borderline tumors (borderline group), 23 ovarian epithelial benign tumors (benign group), and 30 normal ovarian tissue samples (normal group). The median age and age distribution in each group was as follows: ovarian cancer group, 53 years (19-83 years); borderline group, 48 years (26-58 years); benign group, 46 years (24-65 years); and normal group, 45 years (36-67 years). There was no significant difference in age among the groups (*P* > 0.05). The histological types, pathological grades, surgical pathological staging (according to the International Federation of Gynecology and Obstetrics (FIGO), 2006 standard), and lymph node metastasis statuses are shown in Table 1. All specimens were primary lesions with complete clinicopathological data, and no radiotherapy or chemotherapy was performed before surgery. The study was approved by the Ethics Committee of Shengjing Hospital affiliated to China Medical University.

### 2.2. Immunohistochemistry

The ovarian tissues of each group were serially sectioned at 5 *μ*m, and BMPER expression was detected by the immunohistochemical streptavidin-peroxidase-linked (SP) method. BMPER antigen repair was performed by citrate buffer thermal repair. The working dilution of anti-human BMPER antibody (rabbit polyclonal to BMPER, Abcam, USA) was 1 : 75, and the staining method was carried out according to the SP kit instructions (MXB, China).

All the sections were scored under a light microscope in five randomly selected fields at 400-fold magnification. The criteria were based on the staining of the tumor cell membrane and the cytoplasm with brown-yellow granules. All the sections were scored according to the methods described in a previous study [13]. The slides were scored by two senior pathologists who reviewed the slides independently.

### 2.3. Cell Culture and Transfection

Human ovarian cancer cell lines (CAOV3, OVCAR3, SKOV3, and ES-2) were purchased from the Institute of Biochemistry and Cell Biology, Chinese Academy of Sciences (Shanghai, China). CAOV3, OVCAR3, and SKOV3 cells were incubated in 37°C and 5% CO2 incubator with RPMI 1640 medium containing 10% fetal bovine serum (Thermo Fisher Scientific, Waltham, MA, USA), and ES-2 cells were cultured in the same incubator in McCoy's 5A medium containing 10% fetal bovine serum. BMPER was expressed in four ovarian cancer cell lines: CAOV3, OVCAR3, SKOV3, and ES-2. Among these cell lines, the miRNA and protein expression levels of BMPER were higher in CAOV3 and OVCAR3 cells, so these two cell lines were selected for the RNA interference experiment. CAOV3 and OVCAR3 cells were transfected with BMPER-specific siRNA and negative control (NC) using Lipofectamine 3000 (Thermo Fisher Scientific, USA). Cells were collected 72 hours after transfection, and the effects on BMPER interference were detected by RT-PCR and Western blot. The BMPER siRNA sequence (GenePharma, Shanghai, China) is as follows: sense, GGUCCUGUGUGACAGACAUTT and antisense, AUGUCUGUCACACAGGACCTT.

### 2.4. Quantitative Real-Time (RT) PCR

Total RNA from ovarian carcinoma cells was extracted using TRIzol reagent and reverse transcribed into cDNA using an RT-qPCR kit (Takara, Kusatsu, Japan) according to the manufacturer's instructions. PCR was then performed in a 20 *μ*L reaction system containing 2.0 *μ*L of cDNA template. The BMPER forward primer sequence was 5′-TTGTGTTCTACGCCAGTGCC3′, the BMPER reverse primer sequence was 5′-GCAGCACATTCCCAGATGCT-3′, and GAPDH served as an internal reference. An Applied Biosystems® 7500 fast PCR instrument was used for real-time PCR amplification. The reaction cycle parameters were as follows: 40 cycles of denaturation at 95°C for 30 seconds, annealing at 95°C for 5 seconds, and extension at 60°C for 30 seconds. The expression level of the target gene was calculated using the comparative 2^-*ΔΔ*CT^ method.

### 2.5. Western Blot Analysis

Total protein was extracted from cells of each group using RIPA buffer. Denatured proteins were separated by SDS-PAGE through a 10% gel and then transferred to a PVDF membrane. The membrane was blocked with 5% milk or BSA for 2 hours and incubated with the appropriate primary antibody at 4°C overnight. The working concentrations of each antibody were as follows: anti-BMPER (1 : 500; Abcam, USA); anti-mechanistic target of rapamycin (mTOR), anti-phospho-mTOR, anti-LC3A/B, anti-Beclin-1, anti-PCNA, and anti-phospho-MEK 1/2 (1 : 1,000; CST, USA); anti-ERK1/2 (1 : 500; Bioss, Beijing); anti-phospho-ERK 1/2 (1 : 300; Bioss, Beijing); anti-MEK1/2 (1 : 300; Santa Cruz Biotechnology Inc.); anti-Bcl2, anti-Bax, anti-MMP2, anti-MMP9 (1 : 1,000; Proteintech, Rosemont, PA, USA); and anti-GAPDH (1 : 2,000; ZSGB- BIO, Beijing, China). Goat anti-rabbit or goat anti-mouse secondary antibody (1 : 3,000; ZSGB-BIO) was separately added and incubated for 2 hours after the membranes were rinsed with TBST. The protein bands were detected on a GDS8000 gel electrophoresis image analyzer (Thermo Fisher Scientific) using an enhanced electrochemiluminescence (ECL, Thermo Fisher Scientific) detection kit.

### 2.6. Cell Proliferation Assay

Cell proliferation was monitored using the thiazolyl blue tetrazolium bromide assay (MTT). Cells were seeded at a density of 2,000 cells/well in 96-well culture plates. The cell proliferation was monitored every 24 hours from 0 hour to 96 hours. The cells from each well were treated with MTT reagent at 20 *μ*L/well 4 hours prior to harvest. The medium in all wells was removed, and 150 *μ*L of DMSO was added to each well. After 10 minutes of shaking, the OD at 490 nm was measured using a microplate reader.

### 2.7. Apoptosis Assay by Flow Cytometry

Each group of cells was digested with EDTA-free trypsin. After digestion and washing with cold PBS, cells were suspended in 500 *μ*L of Annexin V Binding Buffer (Annexin V, FITC, Dojindo, Japan). Next, 5 *μ*L of Annexin V-FITC solution and 5 *μ*L of PI solution were added to the cell suspension and incubated for 15 minutes in the dark. Apoptotic cells were detected by flow cytometry.

### 2.8. Wound Healing Assay

Cells were seeded into a six-well culture plate, cultured, and then transfected. A 200 *μ*L pipette tip was used to make a linear scratch wound on the well plate. After washing twice with PBS, cells were cultured with FBS-free medium. The width of the scratches was observed under a microscope at 0, 24, and 48 hours after scratching. The area of the scratches was measured with ImageJ, and the wound healing rate was calculated.

### 2.9. Transwell Invasion Assay

For Transwell invasion assay, Matrigel (BD Biosciences, USA) was diluted 1 : 8 with serum-free medium and added to the upper chamber of a Transwell chamber (80 *μ*L/chamber, Corning Incorporated, Corning, NY, USA) on ice. After the Matrigel solidified, 200 *μ*L (cell concentration: 2 × 10^5^ cells/mL) of cells suspended in serum-free medium was added to each upper chamber, and 500 *μ*L of medium containing 10% fetal bovine serum was added to the lower chamber. After the cells were incubated for 24 hours, the membranes were fixed with 4% paraformaldehyde for 30 minutes and stained with crystal violet for 30 minutes. The upper chamber was wiped with a cotton swab. The number of invading cells in 5 fields was counted under a microscope, and the mean value was obtained.

### 2.10. Statistical Analysis

All data were analyzed using the SPSS 24.0 statistical software. Unpaired two-tailed Student's *t*-test and chi-square test were used for comparisons between two groups, and comparisons of differences among more than two groups were analysed by one-way ANOVA. The Kaplan-Meier test was used to produce the survival curve, and the Cox model was used to analyze the relationship of key variables with prognosis. *P* < 0.05 was considered statistically significant.

## 3. Results

### 3.1. BMPER Expression in Different Ovarian Tissues

**Figure 1:**
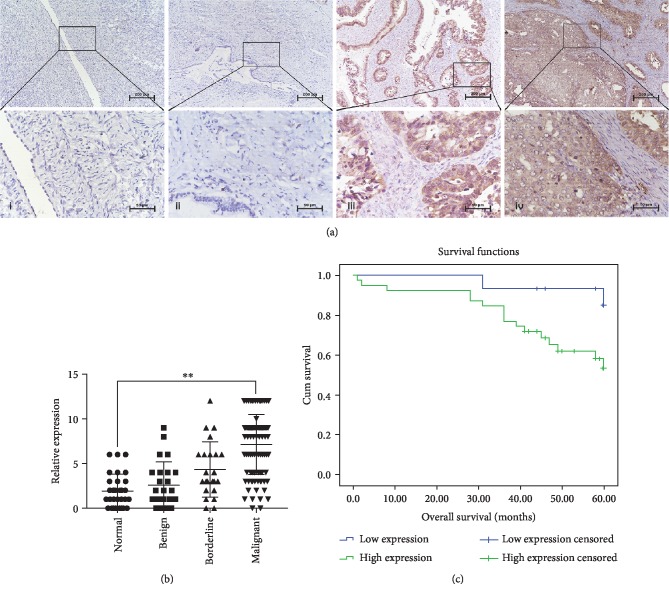
The expression of BMPER protein in different ovarian tissues and its effect on overall survival of ovarian cancer patients. (a) BMPER protein expression in different ovarian tissues (SP, 100×; bottom: 400×) (*n* = 171). The ovarian tissues of each group were serially sectioned at 5 *μ*m, and BMPER expression was detected by the immunohistochemical SP method. All sections were scored under a light microscope in five randomly selected fields at 400× magnification. (i) Normal ovarian tissue (*n* = 30); (ii) ovarian benign tumor (*n* = 24); (iii) ovarian borderline tumor (*n* = 23); and (iv) ovarian epithelial malignant tumor (*n* = 94). (b) Statistical analysis of the results in (a). (^∗∗^*P* < 0.01). (c) Kaplan-Meier survival analysis showed the influence of BMPER expression on the overall survival of ovarian cancer patients; log-rank test *P* = 0.013 for BMPER expression in overall survival (*n* = 94).

BMPER was mainly stained in cytoplasm and intercellular space, and a small number of nuclei were colored with brown or brownish yellow granules. The positive rate and high expression rate of BMPER in ovarian epithelial malignant tumors were 90.43% and 73.91%, respectively, which were significantly higher than the corresponding values in ovarian epithelial borderline tumors (73.91%, 39.13%), ovarian epithelial benign tumors (41.67%, 16.67%), and normal ovarian tissue (30.00%, 10.00%) (*P* < 0.01). Meanwhile, the rate of positive BMPER expression in ovarian epithelial borderline tumors was higher than that in ovarian epithelial benign tumors and normal ovarian tissues (*P* < 0.05), and its high expression rate was higher than that in normal ovarian tissues (*P* < 0.05) (Table 2 and Figures 1(a) and 1(b)).

### 3.2. Relationship between BMPER Expression and Clinicopathological Parameters of Ovarian Cancer

All 94 patients with ovarian cancer in this study had histologically confirmed ovarian epithelial malignancies. High BMPER expression was associated with lymph node metastasis, and the proportion of patients with high BMPER expression in the lymph node metastasis positive group was higher than that in the lymph node metastasis negative group (*P* < 0.05). In terms of pathological types, the rate of high BMPER expression in patients with clear cell carcinoma was higher than that in other pathological types, but it was not statistically significant. There was no difference in BMPER expression among the different FIGO stages and tumor differentiation grades (Table 1).

### 3.3. Associations between BMPER Expression and Prognosis of Patients

There was no significant difference in demographic among the groups. All 94 patients with ovarian cancer were followed up until September 2018. The Kaplan-Meier survival analysis showed that the BMPER expression level was closely related to the overall survival (OS) of patients. The survival rate of patients with high BMPER expression was significantly lower than that of patients with low BMPER expression. Cox regression analysis showed that high BMPER expression was an independent risk factor for poor prognosis in patients (*P* = 0.026). Additionally, the results showed that FIGO stage and lymph node metastasis were also associated with poor prognosis, while tumor differentiation was not correlated with the patient survival (Figure 1(c) and Table 3).

### 3.4. BMPER Expression in Different Ovarian Cancer Cell Lines and Changes in BMPER Expression Levels in CAOV3 and OVCAR3 Cells after siRNA Transfection

**Figure 2:**
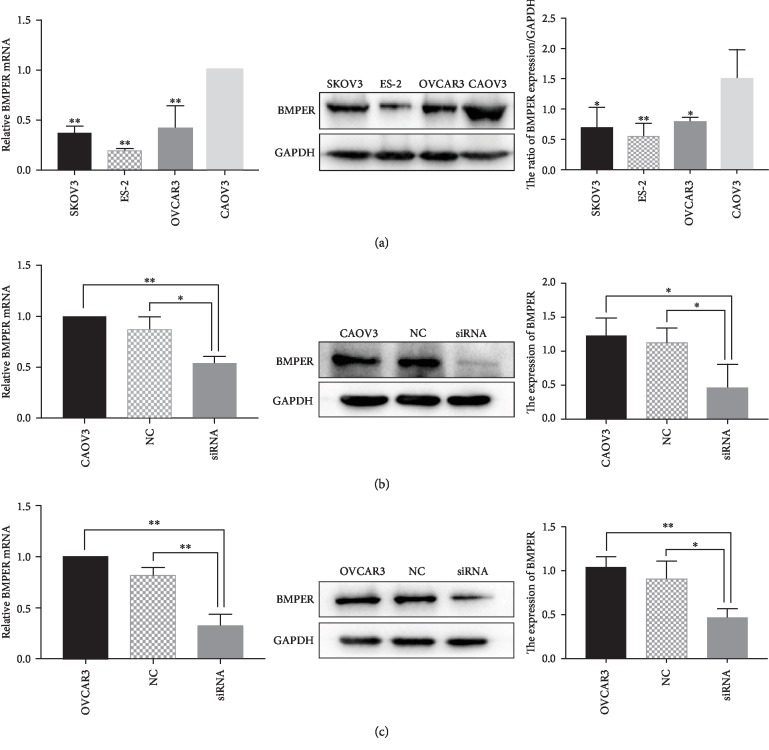
The expression of BMPER in ovarian cancer cell lines. (a) The levels of BMPER mRNA and protein in four ovarian cancer cell lines were detected by real-time PCR and Western blot. ^∗^*P* < 0.05, ^∗∗^*P* < 0.01. (b–c) The expression of BMPER mRNA and protein in ovarian cancer cell lines CAOV3 and OVCAR3 after BMPER siRNA transfection for 72 hours by real-time PCR and Western blot. The asterisks indicate significant differences, ^∗^*P* < 0.05, ^∗∗^*P* < 0.01.

The expression levels of BMPER in four ovarian cancer cell lines CAOV3, OVCAR3, SKOV3, and ES-2 were detected by real-time PCR and Western blot. The results showed that the mRNA and protein expression levels of BMPER in CAOV3 and OVCAR3 cells were higher than those in SKOV3 and ES-2 cells, with ES-2 cells exhibiting the lowest levels of BMPER expression (Figure 2(a)). BMPER siRNA was transfected into CAOV3 and OVCAR3 cells, and BMPER expression was detected after transfection for 72 hours. The results showed that the mRNA and protein expression levels of BMPER in the transfected siRNA group were significantly lower than those in the control and untransfected groups (Figures 2(b) and 2(c)).

### 3.5. Effect of Knocking Down of BMPER Gene Expression on Malignant Biological Behaviors of Ovarian Cancer Cell Lines

**Figure 3:**
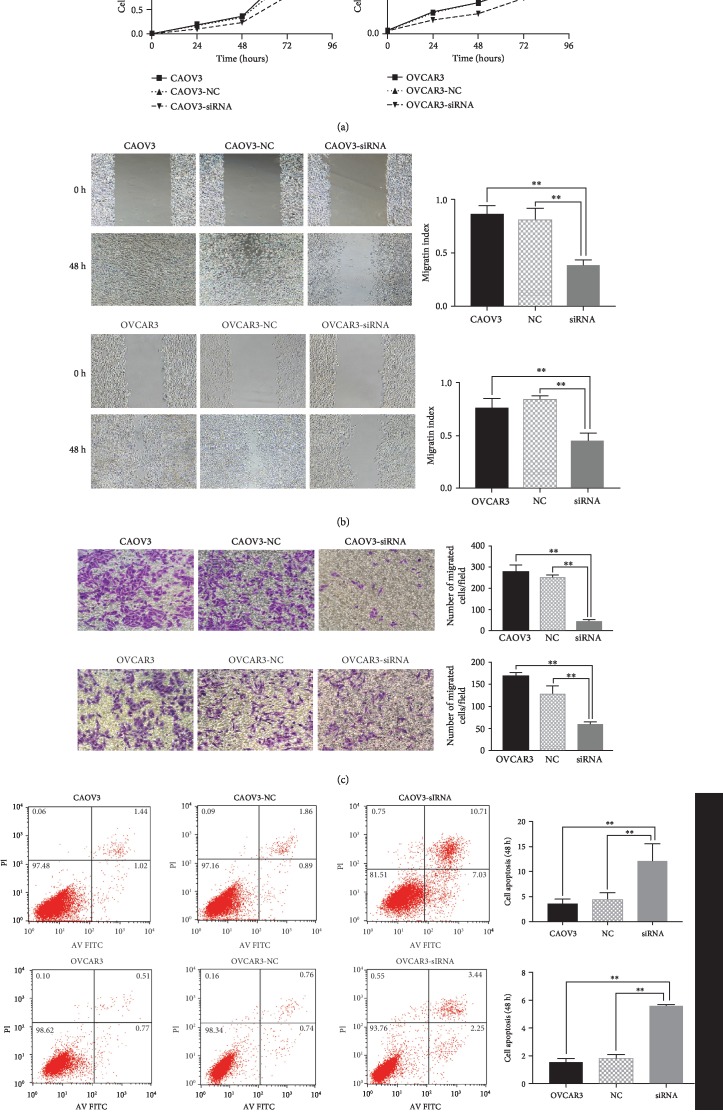
Effects of BMPER specific siRNA transfection on proliferation, migration, invasion, and apoptosis of ovarian cancer cell lines CAOV3 and OVCAR3. Changes in the above malignant behaviors of ovarian cancer cells were detected by MTT, wound healing, Transwell, and flow cytometry assays, respectively. Results showed the inhibition of BMPER with siRNA transfection reduced the proliferation (a), migration (b), invasion (c), and promoted apoptosis (d) of CAOV3 and OVCAR3 cells compared with the control groups. ^∗^*P* < 0.05, ^∗∗^*P* < 0.01.

**Figure 4:**
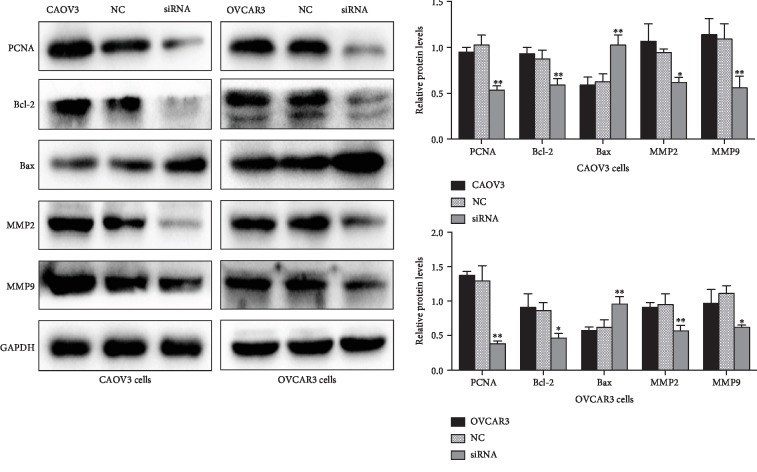
Changes in the expression of PCNA, Bax, Bcl-2, MMP2, and MMP9 in CAOV3 and OVCAR3 cells after transfection of BMPER siRNA. Western blot showed knock down of BMPER gene by siRNA reduced the expressions of PCNA, Bcl-2, MMP2, and MMP9, while the level of proapoptotic protein Bax was increased after that. GAPDH was used as the internal control. Compared with the control group, ^∗^*P* < 0.05, ^∗∗^*P* < 0.01.

After transfection of BMPER-specific siRNA into the ovarian cancer cell lines CAOV3 and OVCAR3, the changes in cell proliferation, migration, invasion, apoptosis, and cell cycle progression were further examined. The expression of proliferating cell nuclear antigen (PCNA), the apoptosis-related indicators B-cell lymphoma 2 (Bcl-2) and Bax, and the invasion- and migration-related indicators matrix metallopeptidase 2 (MMP2) and matrix metallopeptidase 9 (MMP9) were detected by Western blot. The results showed that the proliferation, invasion, and migration abilities of cells transfected with BMPER siRNA were significantly reduced compared with those of the control group, while the apoptosis rate increased (Figure 3); however, the cell cycle distribution did not change significantly (results not shown). In addition, compared with the control group, the siRNA transfection group exhibited decreased expression of PCNA, decreased expression of Bcl-2, increased expression of the proapoptotic protein Bcl-2 family activating protein-X (Bax), and decreased expression of MMP2 and MMP9 (Figure 4).

### 3.6. Downregulation of BMPER Results in Changes in Molecules Related to the MAPK and Autophagy-Related Signaling Pathways

**Figure 5:**
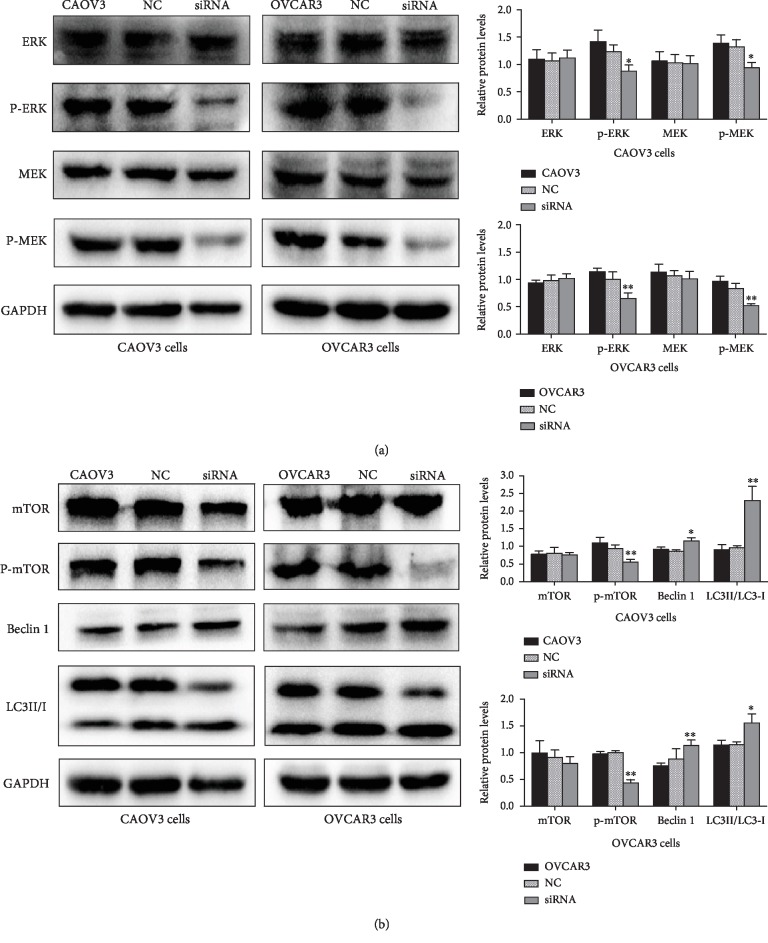
Effect of BMPER siRNA transfection on MAPK and autophagy-related signaling pathways in CAOV3 and OVCAR3 cells. The levels of the MAPK signaling pathway molecules ERK, p-ERK, MEK, and p-MEK and the autophagy-related pathway molecules mTOR, p-mTOR, Beclin 1, and LC3 were detected by Western blot after transfection. GAPDH was used as the internal standard. Inhibition of BMPER reduced the phosphorylation of MAPK signaling pathway-associated proteins (p-ERK, p-MEK) (a), but promoted the expression of autophagy-related signaling pathway molecules (b). ^∗^*P* < 0.05, ^∗∗^*P* < 0.01.

To further understand the mechanism by which downregulation of BMPER causes changes in the biological behavior of ovarian cancer cells and to determine the possible signaling pathway through which BMPER may play a role, siRNA transfection was used to inhibit BMPER expression in CAOV3 and OVCAR3 cells. Subsequently, the expression levels of the MAPK signaling pathway molecules ERK, p-ERK, MEK, and p-MEK and the autophagy-related pathway molecules mTOR, p-mTOR, Beclin 1, and microtubule-associated proteins 1A/1B light chain 3 (LC3) were detected by Western blot analysis. The results showed that after BMPER expression was downregulated, the levels of p-ERK, p-MEK, and p-mTOR proteins decreased while Beclin 1 levels and the LC3II/I ratio increased, indicating that the MAPK signaling pathway was inhibited after interference with BMPER expression but that autophagy-related pathways were promoted upon BMPER knockdown (Figures 5(a) and 5(b)).

## 4. Discussion

BMPER is a homologous protein of Drosophila crossveinless-2 in vertebrates and is expressed in endothelial progenitor cells. The human BMPER gene is located on chromosome 7p14.3. BMPER can affect biological behaviors such as differentiation, proliferation and migration of vascular endothelial cells, regulate angiogenesis, and participate in the development of various organs such as bone, lung, kidney, and some associated diseases [6, 14–16]. Our team used a gene chip to find that BMPER expression was related to the expression of tumor biomarker human epididymis protein 4 [17], but the role and mechanism of BMPER in ovarian cancer has not been reported.

Few reports address the role of BMPER in tumors. Bethge et al. [18] found that 58% of the BMPER gene promoter was methylated in B-cell lymphoma, while no methylation occurred in the control samples. In the field of gynecological oncology, research on BMPER is rare. No studies have investigated the role of BMPER in ovarian cancer. Heinke et al. reported that BMPER can promote biological behaviors such as invasion and migration of cervical cancer cells [7]. The results of this study indicated that the percentages of positive and high BMPER expression in ovarian cancer were significantly higher than other ovarian tissues. The rate of high BMPER expression in clear cell carcinoma was higher than that in other pathological types, but the difference was statistically nonsignificant, probably because of the limited number of samples. The expression level of BMPER was closely related to OS of patients and high BMPER expression was an independent risk factor for poor prognosis. Our results also showed that the inhibition of BMPER expression reduced the proliferation, migration, and invasion of ovarian cancer cell lines CAOV3 and OVCAR3. Although BMPER expression in ES-2 cells is relatively lower than that in other ovarian cancer cells, BMPER may still play a role in promoting the malignant biological behaviors such as invasion and migration of ES-2 cells. In addition, BMPER has been reported to promote fibroblast invasion and migration [16]. The results were consistent with previously reported results of BMPER in other cancers. The above results show that BMPER may promote the occurrence and progression of ovarian cancer and ultimately have an adverse effect on the prognosis of patients with ovarian cancer.

According to the literature, BMPER can function through several signaling pathways in the body. BMPER is a regulator of angiogenesis in the BMP signaling pathway and interacts with BMP2, BMP4, BMP10, etc., playing a concentration-dependent positive and negative bidirectional regulatory role [19–22]. In addition, BMPER can also function through other signaling pathways independent of BMP. Studies have shown that BMPER promotes angiogenesis by increasing the expression and activity of the constituents of the fibroblast growth factor (FGF) signaling pathway and inhibiting the anti-angiogenic factor thrombospondin-1 [23]. Esser et al. found that BMPER plays an important role in maintaining normal arteriovenous morphology and preventing vascular malformation in the embryo as a necessary factor to maintain the activity of the Notch signaling pathway in endothelial cells [24]. To understand the molecular mechanism underlying BMPER's role in ovarian cancer, the possible signaling pathways were detected after BMPER siRNA transfection in this study, and it was later proven that the levels of the phosphorylated proteins p-ERK and p-MEK in the MAPK signaling pathway were reduced. This study confirmed for the first time that BMPER could affect the malignant biological behavior of ovarian cancer cells through the MAPK signaling pathway. Currently, some MAPK pathway inhibitors, including selumetinib, SB203580, and PD98059, have been used in basic and clinical trials of ovarian cancer. However, the use of MAPK pathway inhibitors alone has drug resistance problems and can lead to the activation of the bypass pathways; thus, MAPK pathway inhibitors are often used in combination with other drugs in clinical practice [25–27].

In addition, the expression level of autophagy-related molecules was found changed after the inhibition of BMPER expression, which affected the autophagy of ovarian cancer cells. mTOR is a serine/threonine kinase in the phosphatidylinositol 3-kinase-related protein kinase family and plays a negative regulatory role in the process of autophagy by inhibiting it. Beclin 1 is a proven autophagy-associated protein that promotes autophagy and plays a tumor-suppressive role in the lysosomal degradation pathway of autophagy [28]. Our previous experiments confirmed that patients with higher Beclin 1 expression had a better prognosis than those with lower Beclin 1 expression [29]. LC3 is a key protein involved in autophagy and an important marker for monitoring this process. When LC3 is lipidated from water-soluble type I to type II, it marks the occurrence of autophagy. When mTOR is inhibited, Beclin 1 is activated, the conversion of LC3 I to LC3 II increases, and autophagy is increased, resulting in degradation and recycling of cellular components [30]. In this study, we found that inhibition of BMPER expression resulted in decreased p-mTOR levels and increased Beclin 1 expression and conversion of LC3 I to LC3 II. These changes promote cell autophagy. These results demonstrate that BMPER is involved in the regulation of autophagy in ovarian cancer cells and inhibits autophagy, which may promote the malignant biological behavior of ovarian cancer cells and the progression of ovarian cancer.

There are some limitations in this study. Because BMPER is over expressed in ovarian cancer tissues and cells, we have only performed BMPER knockdown experiments and conducted limited research on the molecular mechanism of pathways. The gain-of-function experiment was not carried out in normal ovarian cells. In addition, the immuno-co-staining experiment may be more helpful in determining the type of cells expressing BMPER in epithelial ovarian tumors. However, the current results of immunohistochemistry, cellular biological experiments, and pathway mechanism after knockdown of BMPER expression have important value in clarifying the role of BMPER in ovarian cancer. In the follow-up studies, we will continue improving the experiment and further study the molecular regulatory mechanism of BMPER on ovarian cancer.

## 5. Conclusion

This study demonstrates for the first time that BMPER is highly expressed in ovarian cancer and is an independent risk factor for prognosis. BMPER may play important roles in the occurrence and progression of ovarian cancer as both a potential prognostic marker and as a new strategy for the treatment of ovarian cancer.

## Figures and Tables

**Table 1:** Relationships between BMPER expression and clinicopathological characteristics of 94 patients with epithelial ovarian cancer.

Characteristics	*n*	Low		High		High expression rate (%)	*P*
		(-)	(+)	(++)	(+++)		
FIGO stage							>0.05
I-II	41	4	9	8	20	68.29	
III-IV	53	6	5	14	24	71.70	
Differentiation							>0.05
Well-moderate	49	5	9	12	23	71.43	
Poor	45	4	9	11	21	71.11	
Pathologic type							>0.05
Serous	59	7	12	12	28	67.80	
Mucinous	10	2	1	3	4	70.00	
Endometrioid	17	0	4	2	11	76.47	
Clear cell carcinoma	8	0	1	3	4	87.50	
LN metastasis							0.044^∗^
No	56	6	14	14	22	64.29	
Yes	23	1	2	5	15	86.96	
No lymphadenectomy	15	2	2	3	8	73.33	

Note: ^∗^*P* < 0.05.

**Table 2:** The expression of BMPER in different ovarian tissues.

Groups	Cases	Low high				Positive rate (%)	High expression rate (%)
		(-)	(+)	(++)	(+++)		
Malignant	94	9	18	22	45	90.43^∗^	71.27^∗∗^
Borderline	23	6	8	6	3	73.91^∗^	39.13
Benign	24	14	6	3	1	41.67	16.67
Normal	30	21	6	3	0	30.00	10.00

Note: ^∗^*P* < 0.05, and ^∗∗^*P* < 0.001.

**Table 3:** Multivariate Cox regression analysis of prognosis.

Variables	Hazard ratio (95% CI)	*P* value
FIGO stage	5.908 (1.313-26.583)	0.021^∗^
Differentiation	1.712 (0.644-4.554)	0.281
Lymph node metastasis	4.038 (1.006-16.210)	0.036^∗^
BMPER	5.522 (1.230-24.789)	0.026^∗^

Note: ^∗^*P* < 0.05.

## Data Availability

The data used to support the findings of this study are available from the corresponding author upon request.
